# Elevated high sensitivity cardiac troponin T among men with HIV who use stimulants: A cross-sectional study of subclinical cardiovascular injury

**DOI:** 10.1371/journal.pone.0350086

**Published:** 2026-06-08

**Authors:** Michaela E. Larson, Yue Pan, Lisa Reidy, Emily M. Cherenack, Sabina Hirshfield, Keith J. Horvath, Elise D. Riley, Savita Pahwa, Suresh Pallikkuth, Claudia Martinez, Adam W. Carrico

**Affiliations:** 1 Department of Epidemiology, School of Public Health and Social Work, Florida International University, Miami, Florida, United States of America; 2 Department of Public Health Sciences, University of Miami Miller School of Medicine, Miami, Florida, United States of America; 3 Department of Pathology and Laboratory Medicine, University of Miami Miller School of Medicine, Miami, Florida, United States of America; 4 Department of Psychiatry and Behavioral Sciences, University of Miami Miller School of Medicine, Miami, Florida, United States of America; 5 Department of Medicine, STAR Program, State University of New York Downstate Health Sciences University, Brooklyn, New York, United States of America; 6 Department of Psychology, San Diego State University, San Diego, California, United States of America; 7 Department of Medicine, University of California San Francisco, San Francisco, California, United States of America; 8 Department of Microbiology and Immunology, University of Miami Miller School of Medicine, Miami, Florida, United States of America; 9 Department of Medicine, University of Miami Miller School of Medicine, Miami, Florida, United States of America; 10 Department of Health Promotion and Disease Prevention, School of Public Health and Social Work, Florida International University, Miami, Florida, United States of America; Virginia Commonwealth University, UNITED STATES OF AMERICA

## Abstract

People with HIV (PWH) experience elevated cardiovascular disease risk compared to people without HIV. Stimulant use may further increase subclinical myocardial injury among PWH, but data on cardiovascular biomarkers, including serum high-sensitivity cardiac troponin T (hs-cTnT) in this population is limited. This cross-sectional secondary analysis included 72 cisgender men with and without HIV enrolled in a South Florida cohort. Stimulant exposure was defined as any non-prescribed stimulant use in the past 3 months and/or a reactive urine toxicology screen, creating four HIV-by-stimulant use groups (i.e., HIV+Stim + , HIV+Stim-, HIV-Stim + , and HIV-Stim-). hs-cTnT was measured using a Roche high-sensitivity assay, with values below the limit of detection treated as undetectable. We used a two-part model (logistic for detectability; log-normal among participants with detectable hs-cTnT), adjusted for age and recent tobacco use, with sensitivity analyses adding renal function and cardiometabolic factors. After adjusting for age and recent tobacco use, HIV+Stim+ participants had higher odds of detectable hs-cTnT (aOR = 7.48, 95% CI: 1.25, 44.62) and higher estimated mean concentration of hs-cTnT (β = 0.51, *p* = 0.031, *mean* = 12) than the HIV-Stim- group. Exploratory analyses suggested a positive dose-response association between amphetamine metabolite levels and hs-cTnT (*r*(11) = 0.86, *p* < 0.0001). Co-occurring HIV and stimulant use were associated with higher hs-cTnT in this sample. However, given that hs-cTnT may reflect a range of acute, subacute, and chronic processes, and the small sample size and restricted generalizability, these findings should be interpreted as exploratory and hypothesis-generating and require confirmation in larger studies.

## Introduction

The burden of non-communicable diseases among people with HIV (PWH) is growing [1]. Even in the modern antiretroviral therapy (ART) era, PWH in the United States (US) on effective ART experience shorter life expectancies, and increased rates of diabetes, mental health conditions, certain cancers, and cardiovascular diseases (CVD) [2–4]. The risk for CVD is thought to be approximately 50% higher among PWH compared to individuals without HIV, even when controlling for clinical risk factors and demographic characteristics [5–12]. However, the biobehavioral mechanisms underlying the increased risk of CVD among PWH are still poorly understood. Recent evidence suggests that HIV-specific risk factors such as ART-related side effects, viremia, inflammation, and immune activation may play a role in CVD risk, but findings are inconsistent across studies [13–17].

Substance use is another known risk factor for CVD. Although cocaine is a well-established CVD risk factor, less is known about other stimulants such as methamphetamine. In a recent retrospective cohort study in older adults without HIV there was a 40% increase in cardiovascular events 30 days after the initiation of prescribed stimulants (i.e., amphetamine, methylphenidate, lisdexamfetamine, or dextroamphetamine) [18]. Studies investigating the misuse of prescribed or use of non-prescribed stimulants have also documented increases in cardiovascular events including hypertension, cardiomyopathy, heart failure, myocardial infarction, endocarditis, aortic dissection, and stroke [19–25].

Among PWH, the prevalence of stimulant use is estimated to be between 5–15% [26–29]. A recent nationwide study estimated the prevalence of stimulant use as 9.2%, 7.5%, and 3.2% for gay, bisexual men and heterosexual men, respectively [30]. Stimulant use among PWH is associated with poorer ART adherence and persistence, unsuppressed viral load, greater inflammation, and faster clinical progression [31–35]. An important gap is that relatively few studies have examined whether and how stimulant use could heighten subclinical cardiovascular injury among PWH compared to people without HIV.

Cardiac Troponin (cTn) is a complex of proteins that regulates cardiac muscle contraction and relaxation [36]. Normal cardiac function is dependent on all three subunits – C, T, and I. Cardiac Troponin T (cTnT) is the largest of the three subunits and is expressed as four isoforms [37]. Elevated cTnT has been linked with a wide range of cardiovascular outcomes, including arrythmia, infarction, heart failure, ischemia, injury, and carditis [38,39]. The inclusion of biomarkers such as cTnT in risk models improves the prediction of cardiovascular risk in the general population [40,41]. Since 2017 [42] a growing number of high-sensitivity assays have been developed and allow for the detection of subclinical cardiac injury, and have an approximately 100-fold improvement in analytical sensitivity [43]. Among PWH, high sensitivity cTn (hs-cTn) assays have been used to estimate subclinical injury confirmed by imaging modalities (e.g., cardiac magnetic resonance) with variable results [44–48]. Others have shown a dose-response relationship between the concentration of cocaine metabolites and hs-cTnI concentration among men and women [49], as well as longitudinal associations between cocaine/alcohol co-use and hs-cTnI among women living with and without HIV [50]. Whether similar results would be observed among men living with and without HIV who use amphetamines is unknown.

Conceptually, we posit a biobehavioral model in which HIV-related immune dysregulation and stimulant-related cardiotoxic pathways jointly increase myocardial stress and microvascular injury, resulting in detectable hs-cTnT as an indicator of subclinical cardiac injury. In this cross-sectional secondary analysis, we examined the associations of HIV by stimulant use groups (i.e., HIV+Stim + , HIV+Stim-, HIV-Stim + , and HIV-Stim-) with (1) detectable hs-cTnT and (2) estimated mean hs-cTnT levels. Our primary hypothesis was that HIV+Stim+ participants would have greater odds of detectable hs-cTnT and higher estimated mean levels of hs-cTnT than the other groups. Our secondary hypothesis was that among participants with evidence of recent amphetamine exposure, higher serum amphetamine metabolite concentrations would be associated with higher hs-cTnT. Exploratory analyses examined correlations between hs-cTnT and inflammatory and metabolic biomarkers relevant to CVD.

## Methods

### Study design

This cross-sectional study leveraged baseline self-report and biospecimen from a prospective cohort study focused on estimating the incidence of the novel coronavirus (i.e., SARS-CoV-2). Participants were recruited primarily through advertisements on social networking applications (e.g., Grindr, SCRUFF). Data were collected from August 2020 to February 2022 in Miami-Dade and Broward Counties, Florida. This study enrolled men with and without HIV stratified by stimulant use. Eligible participants were: cisgender men; 18 years of age and older; proficient in English; and reported anal sex with a cisgender man in the past year. All study activities were approved by the University of Miami Institutional Review Board (IRB). Participants completed a written informed consent process prior to initiating study activities, and all procedures were conducted according to the principles expressed in the Declaration of Helsinki. Eligible and consented participants were asked to complete an online survey and attend a single in-person visit to provide urine and peripheral venous blood samples and anthropometric measurements (e.g., height, weight, blood pressure). Study methods have been described previously in detail [51]. Of the 75 men enrolled in the parent study, this secondary analysis included all participants (N = 72) who provided blood samples required for each of the assays discussed below and provided written consent for these samples to be used for future/secondary research purposes. Because hs-cTnT was not a primary outcome for the parent study, no a priori sample-size calculation was performed specifically for the present analyses. However, we report effect sizes with 95% confidence intervals and include a sensitivity analysis describing the minimum detectable effects given the fixed sample size. Given the use of de-identified data for this secondary analysis, and the obtained consent from participants as part of the main study, this analysis was exempt from further review by the IRB. Stored samples were analyzed in August 2023.

## Measures

### Demographics

Participants provided demographic information, including age, race and ethnicity, and sexual orientation.

### Substance use

Individuals who reported any use of non-prescribed stimulants in the last three months via the Alcohol, Smoking, and Substance Involvement Screening Test (ASSIST) or had a reactive iCup Drug Screening Devices [52] result for cocaine or amphetamine were categorized as people who use stimulants (Stim+). Information regarding stimulant and tobacco use in the last three months was collected with a modified version of the World Health Organization’s ASSIST [53]. iCup devices were used to detect cocaine and amphetamine metabolites in urine, indexing any use in the past 72 hours. The iCup captures binary (yes/no) use of any cocaine, methamphetamine, amphetamine (a metabolite of methamphetamine; may also capture prescribed stimulants), marijuana (THC), and benzodiazepine use.

Where participants provided a reactive urine screen for cocaine or amphetamine, we measured metabolites in stored sera samples. The analysis of cocaine metabolites produced unstable results and are therefore not presented. Serum samples were prepared and analyzed using liquid-chromatography mass spectrometry (LC-MS/MS). The stimulants and their deuterated internal standards were extracted from the serum by buffering them to pH 6.0. The metabolites were then isolated from the blood by passing the matrix through the hydrophilic DVB polymer/cation exchange, cross-linked solid-phase extraction column (SPE, Cerex Trace B, 35 mg). The columns were then washed, and amphetamine and methamphetamine were eluted and collected.

After evaporation, the residues containing amphetamine and methamphetamine were reconstituted with the mobile phase, and the extract was injected on the LC-MS/MS (Agilent Technologies Santa Clara, CA, USA) instrument operating in MRM mode. This method is validated according to ASB 006 guidelines, including linearity (5–500 ng/mL), the lower limit of detection (LOD – 1 ng/mL), accuracy, ionization suppression/enhancement, carryover, stability, and interference. In this study, we focused on amphetamine metabolites because methamphetamine is rapidly metabolized to amphetamine following use.

### HIV status

HIV-negative serostatus was confirmed with the OraQuick ADVANCE Rapid HIV-1/2 Antibody Test [54]. Among PWH, plasma samples were used to measure HIV-1 viral load. Undetectable viral load was defined as < 20 copies/mL [55].

### Metabolic factors

Relevant metabolic factors were examined to better index cardiovascular-related health. Height (in inches), weight (in pounds), and blood pressure were measured. Blood pressure measurements were categorized into hypertension categories: no hypertension (systolic < 120 mm HG and diastolic < 80 mm HG), elevated (systolic > 120 mm Hg and diastolic < 80 mm Hg), stage 1 hypertension (systolic ≥ 130 mm Hg or diastolic ≥ 80), stage 2 hypertension (systolic ≥ 140 mm Hg or diastolic ≥ 90 mm Hg), hypertensive crisis (systolic ≥ 180 or diastolic ≥ 120).

Non-fasting plasma samples were used for a routine lipid panel, including triglyceride, total cholesterol, and high- and low-density lipoprotein (HDL and LDL) cholesterol concentrations, and a comprehensive metabolic panel to assess insulin and glucose concentrations, and kidney and liver function (e.g., creatinine). Individuals with triglyceride levels ≥150 mg/dL, total cholesterol ≥200 mg/dL, LDL cholesterol ≥130 mg/DL, or HDL cholesterol <40 mg/dL were categorized as having dyslipidemia. Insulin (mU/L) and glucose (mg/dL) concentration were used to calculate non-fasting Homeostatic Model Assessment of Insulin Resistance (HOMA-IR) scores.

### Markers of inflammation

Given that inflammation may mediate the relationship between stimulant use and cardiovascular disease, several markers of inflammation were examined in the sample. They were included in this analysis to explore their relationship with hs-cTnT. Soluble CD163 (sCD163), interleukin 6 (IL-6), tumor necrosis factor receptor 1 and 2 (TNFRI, TNFRII), intracellular adhesion molecule 1 (ICAM-1), and trimethylamine N-oxide (TMAO) were measured in plasma using Luminex bead based multiplex analysis or enzyme-linked immunosorbent assay (ELISA). High-sensitivity c-reactive protein (hs-CRP) was measured in serum using ELISA with a detection limit of 0.000352 mg/L. hs-CRP values ≥1 mg/L were categorized as elevated [56–58], and all others were categorized as normal.

### SARS-CoV-2

Recent/current SARS-CoV-2 infection was operationalized as SARS-CoV-2 IgM serostatus (positive or negative).

### High sensitivity cardiac Troponin T

Serum hs-cTnT concentrations were analyzed via Roche Diagnostics’ Elecsys high-sensitivity assay, with a lower limit of detection of 5 ng/L or 0.005 ng/mL. Participants with concentrations below the limit of detection were treated as undetectable for a binary variable (detectable/undetectable). For continuous components, aside from descriptive statistics, hs-cTnT was modeled on the log scale among detectable values only (i.e., values below the limit of detection were missing for part 2 of the model described below). Due to prior research showing an association between HIV and stimulant use and hs-CRP in this sample, we followed a multimarker strategy [59] to create a combined troponin and hs-CRP risk stratification variable that categorized participants based on the number of elevated biomarkers. Participants with a detectable troponin and hs-CRP ≥1 were categorized as both elevated. Those with only one of these were categorized as one elevated. Participants with a hs-CRP ≤1 and undetectable troponin were categorized as neither elevated.

### Analysis

Descriptive statistics were calculated to characterize the sample and assess outliers, departures from normality, or missing data issues. Kruskal-Wallis, Chi-Square, and Fisher’s Exact tests were used to examine differences in participant characteristics across HIV by stimulant use groups. Exploratory, hypothesis-generating associations between continuous hs-cTnT with amphetamine metabolite concentrations and inflammatory biomarkers were analyzed using bivariate Pearson correlations. These analyses were conducted to examine dose-response associations of methamphetamine or amphetamine use. Given the modest sample size, limited power to detect small-to-moderate associations, and the exploratory nature of these correlations, we did not apply formal multiple-comparison adjustment.

Hs-cTnT was zero-inflated with many participants displaying undetectable levels. To accommodate this, we utilized a two-part technique to simultaneously model both the predicted probability of a detectable hs-cTnT (logistic portion of model), and the estimated mean level of hs-cTnT (log-normal portion of model) across HIV by stimulant use groups. For the log-normal portion of the model, we assumed non-constant variances for all continuous variables given results of Levene’s tests and examination of standardized residuals. Final model selection was guided by results from the univariate models, known hs-cTnT covariates, model Akaike Information Criterion (AIC), and sample size. The final model was selected based on the lowest AIC value. Covariate selection was guided by an a priori causal framework, with the goal of estimating the association of HIV by stimulant groups with hs-cTnT while avoiding overfitting/overadjustment. For covariates that did not make it into the final model, but may lie on the causal pathway, we evaluated them in sensitivity analyses. We also conducted a pos-hoc sensitivity analysis using a stricter stimulant definition based on the ASSIST involvement scores for cocaine and methamphetamine (scores > 3 classified as stimulant use). There were no missing data across key demographic and clinical characteristics. Individuals with missing data from variables used in the exploratory Pearson correlations (amphetamine concentration, creatinine, TNF) were excluded. All tests had a two-tailed significance with alpha set at 0.05, and analyses were performed using SAS Version 9.4.

Given that this was a secondary analysis with a fixed sample size (N = 72), we conducted a power assessment (two-sided α = 0.05) to characterize the magnitude of effects that could be detected with 80% power. For the omnibus four-group comparison, the minimum detectable standardized effect was large (Cohen’s f = 0.40, η^2^ = 0.14). For pairwise group mean differences, detectable standardized differences were approximately d = 1.0. For the detectable/undetectable (logistic) component of our model, the available sample size provides approximately 80% power for large differences in detectability, corresponding to an increase from about 33% to 79% in the proportion with detectable hs-cTnT. Finally, because the log-normal component of our model is estimated among participants with detectable hs-cTnT only, the effective sample size per group is smaller. Thus, detectable effects are very large (Cohen’s f = 0.63). The findings presented here should be interpreted as exploratory and are most sensitive to large between-group differences, particularly for the analyses restricted to detectable hs-cTnT.

## Results

Participant demographics as a function of HIV by stimulant use group are presented in Table 1. Almost all participants with HIV (*n* = 23, 82%) had undetectable viral load (< 20 copies/mL) and reported taking ART (*n* = 27, 96%). About half were classified as stimulant users (*n* = 33, 46%). The sample was divided into four groups based on HIV status and stimulant use: HIV+Stim+ (*n* = 13), HIV+Stim- (*n* = 15), HIV-Stim+ (*n* = 20), and HIV-Stim- (*n* = 24). A majority of the sample was racially/ethnically diverse, with 75% of participants identifying as Non-Latino Black/African American or Latino. Eighty-one percent (*n* = 58) identified as gay, and 19% (*n* = 14) identified as bisexual, straight, or other. HIV+Stim+ participants were significantly older on average compared to other groups. The groups did not differ significantly based on recent/current SARS-CoV-2 infection.

**Table 1:** Demographic characteristics and cardiovascular risk indicators among men enrolled from August 2020 – February 2022 (*N* = 72).

Study Characteristic	HIV by Stimulant Use Group				p-value
	HIV+ Stim+ (n = 13)	HIV+ Stim- (n = 15)	HIV- Stim+ (n = 20)	HIV- Stim- (n = 24)	
**Demographics**	n (%)	n (%)	n (%)	n (%)	
**Age, *mean (SD)***	47 (11)	38 (11)	35 (10)	36 (13)	0.011*
*Median (IQR)*	45 (42-53)	35 (29-42)	32 (27-41)	30 (27- 44)	
**Race/Ethnicity**					0.076
Black, Non-Latino	3 (23)	5 (33)	1 (5)	2 (8)	
White, Non-Latino	5 (39)	2 (13)	4 (20)	6 (25)	
Any Race, Latino	4 (31)	8 (53)	15 (75)	16 (67)	
Other, Non-Latino	1 (8)	0 (0)	0 (0)	0 (0)	
**SARS-CoV-2**					
**Recent/Current SARS-CoV-2**					0.342
Yes	1 (8)	0 (0)	3 (15)	1 (4)	
No	12 (92)	15 (100)	17 (85)	22 (96)	
**CV Risk Factors**					
**BMI, *mean (SD)***	31 (9)	27 (6)	25 (6)	27 (6)	0.113
*Median (IQR)*	28 (26, 33)	28 (24, 28)	25 (22, 28)	25 (23, 29)	
**Hypertension**					0.062
None	2 (15)	3 (20)	6 (30)	13 (54)	
Elevated BP	1 (8)	3 (20)	5 (25)	3 (13)	
Stage 1 Hypertension	4 (31)	7 (47)	8 (40)	6 (25)	
Stage 2 Hypertension	6 (46)	2 (13)	1 (5)	2 (8)	
Hypertensive Crisis	–	–	–	–	
**Dyslipidemia** ^a^					0.571
Yes	7 (54)	10 (67)	12 (60)	18 (75)	
No	6 (46)	5 (33)	8 (40)	6 (25)	
**HOMA-IR, *mean (SD***)^a^	5 (6)	4 (6)	6 (8)	5 (4)	0.441
*Median (IQR)*	3 (2, 4)	2 (1, 3)	3 (1, 8)	4 (2, 6)	
**Any Tobacco Use (past 3-months)**					0.001*
Yes	8 (62)	3 (20)	13 (65)	4 (17)	
No	5 (39)	12 (80)	7 (35)	20 (83)	
**Biomarkers**					
**hs-cTnT (ng/L), *mean (SD)***	14 (16)	6 (8)	3 (5)	3 (4)	0.010*
*Median (IQR)*	10 (7, 18)	0 (0, 10)	0 (0, 7)	0 (0, 7)	
hs-CRP*hs-cTnT risk stratification					0.010*
Both elevated	8 (62)	3 (20)	5 (25)	1 (4)	
One elevated	3 (23)	9 (60)	8 (40)	12 (50)	
Neither elevated	2 (15)	3 (20)	7 (35)	11 (46)	

* Significant at p = 0.05; continuous variables analyzed with Kruskal-Wallis; categorical variables analyzed with chi-square and Fisher’s exact test.

^a^From non-fasting blood draws

### HIV by stimulant use groups and cardiovascular risk

Table 1 details information regarding self-report measures and laboratory-confirmed metabolic factors. Overall, the groups had similar distributions across metabolic factors, including BMI, hypertension, dyslipidemia, and insulin resistance. The average BMI across groups was 27 (*SD* = 7), with the highest mean BMI among HIV+Stim+ participants (*M* = 31; *SD* = 9). Seventy-seven percent (77%) of HIV+Stim+ participants had stage 1 (31%) or stage 2 (46%) hypertension. However, hypertension status did not vary significantly across groups. Similarly, the proportion of individuals with dyslipidemia did not vary significantly across the stimulant use and HIV groups. Tobacco use did vary significantly across groups (*p* = 0.001), with HIV+Stim+ (62%) and HIV-Stim+ participants (65%) having the highest proportions of reported tobacco use in the past three months compared to HIV+Stim- (20%) and HIV-Stim- (17%) participants.

### hs-cTnT, & multimarker stratification

As shown in Table 1, hs-cTnT concentration differed significantly across the groups (*p =* 0.010), with HIV+Stim+ participants having the highest concentration compared to all other groups. Multimarker stratification of hs-CRP and troponin indicated that the groups differed significantly in terms of whether they had one or both biomarkers elevated (*p* = 0.010). Overall, 68% of the sample had one or both biomarkers elevated. HIV+Stim+ participants had the highest proportion with both biomarkers elevated.

### Exploratory Associations of stimulant metabolites and markers of inflammation with hs-cTnT

**Fig 1:**
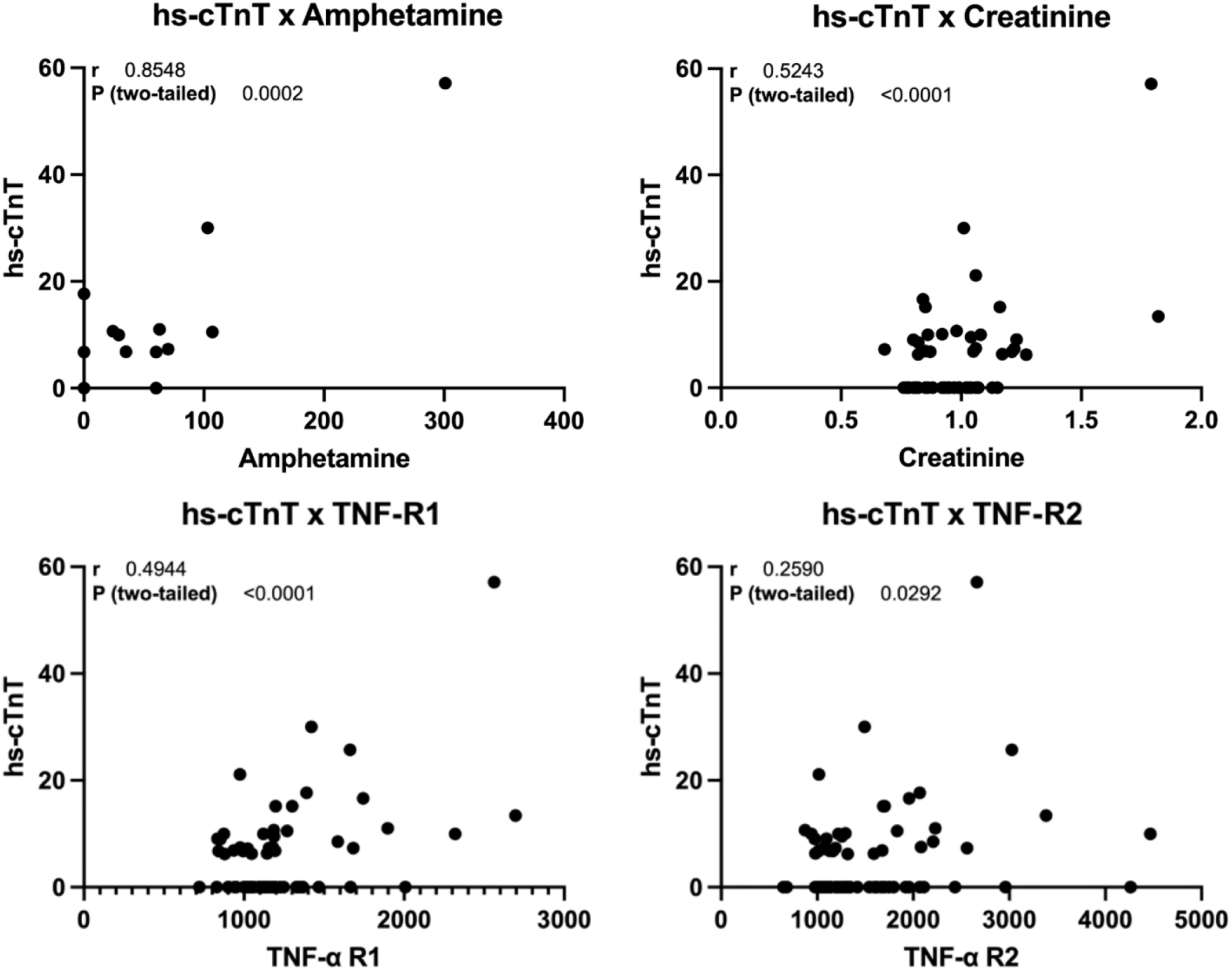
hs-cTnT correlations with substance use metabolites and biomarkers.

Correlations between hs-cTnT with stimulant use metabolites and inflammatory biomarkers are shown in Fig 1a-1d. In a small analytic subset, amphetamine concentration (ng/mL) was positively correlated with hs-cTnT levels (*r*(11) = 0.85, *p* < 0.0001). Given the limited sample size, these findings should be interpreted cautiously as preliminary and potentially sensitive to influential observations. Relevant inflammatory and kidney-related biomarkers that were positively associated with hs-cTnT concentration included: TNFRI (*r*(69) = 0.4944, *p* < 0.0001), TNFRII (*r*(69) = 0.2590, *p* = 0.0292), and serum creatinine (*r*(56) = 0.5243, *p* < 0.0001).

### Two-part Model for hs-cTnT

In the unadjusted models (Table 2), HIV+Stim+ participants had significantly higher odds of having a detectable troponin level (*OR* = 6.7, *95% CI* = 1.4–31.2, p = 0.018) compared to HIV-Stim- participants. HIV+Stim+ participants also had a higher estimated mean hs-cTnT concentration *(mean* = 12, *SD* = 0, *p* = 0.012) than HIV-Stim- participants (*mean* = 3, *SD* = 0). In the adjusted two-part model (Table 3), the association between HIV by stimulant use groups with hs-cTnT was examined while adjusting for any recent tobacco use and age. Adjusting for tobacco use and age, HIV+Stim+ participants were significantly more likely (76.9%, *p = 0.039*) to have a detectable hs-cTnT compared to HIV-Stim- participants (33.3%) in the zero-inflated portion of the model. Furthermore, HIV+Stim+ participants also had a significantly higher estimated mean hs-cTnT concentration (*M* = 12; *SD* = 3; *p* = 0.004) compared to HIV-Stim- participants (*M* = 3; *SD* = 2). In sensitivity analyses, results from models with serum creatinine levels, hypertension category, or recent/current SARS-CoV-2 did not change the direction or magnitude of the group differences in detectable hs-cTnT or mean hs-cTnT. Furthermore, in a sensitivity analysis using a stricter assessment of stimulant use (ASSIST involvement scores for methamphetamine or cocaine greater than 3), the direction and magnitude of adjusted associations were similar to the primary analysis. The detectability (logistic) component was attenuated, while the log-normal component remained similar.

**Table 2:** Unadjusted Two-part regression models (*N* = 72).

Detectable hs-cTnT (Zero-Inflated Portion of Model)			
	Predicted Probability^a^	OR (95% CI)	p
Stimulant use & HIV status			
Stim+HIV+	76.9%	6.667 (1.423, 31.232)	0.018*
Stim+HIV-	35.0%	1.077 (0.308, 3.762)	0.187
Stim-HIV+	46.7%	1.750 (0.466, 6.568)	0.871
Stim-HIV- (ref)	33.3%	–	–
Hypertension			
None (ref)	37.5%	–	–
Elevate BP	50.0%	1.667 (0.410, 6.767)	0.904
Stage 1 Hypertension	32.0%	0.784 (0.241, 2.549)	0.051
Stage 2 Hypertension	81.8%	7.500 (1.315, 42.762)	0.020*
Age	44.4%	1.054 (1.009, 1.100)	0.017*
Tobacco use (past 3-months)			
Yes	49.2%	0.709 (0.271, 1.854)	0.483
No (ref)	41.4%	–	–
hs-cTnT Concentration (Log-Normal Portion of Model)^b^			
	Estimated Mean (SD) hs-cTnT (ng/L)	Model Effects (SE)	p
Stimulant use & HIV status			
Stim+HIV+	12 (0)	0.580 (0.215)	0.012*
Stim+HIV-	4 (0)	0.112 (0.235)	0.638
Stim-HIV+	6 (0)	0.328 (0.235)	0.174
Stim-HIV- (ref)	3 (0)	–	–
Hypertension			
None (ref)	4 (1)	–	–
Elevate BP	6 (1)	0.346 (0.163)	0.191
Stage 1 Hypertension	4 (0)	0.345 (0.238)	0.158
Stage 2 Hypertension	9 (1)	0.224 (0.231)	0.340
Age	5 (3)	0.013 (0.007)	0.056
Tobacco use (past 3-months)			
Yes	5 (0)	−0.053 (0.190)	0.780
No (ref)	6 (0)	–	

^a.^Probability of detectable hs-cTnT; ^b.^ Detectable values only

**Table 3:** Adjusted two-part regression model examining the adjusted associations of HIV by stimulant use goups with hs-cTnT (*N* = 72).

Detectable hs-cTnT (Zero-Inflated Portion of Model)			
	Predicted Probability^a^	aOR (95% CI)	p
Stimulant use & HIV status			
Stim+HIV+	76.9%	7.475 (1.252, 44.620)	0.039*
Stim+HIV-	35.0%	1.770 (0.420, 7.457)	0.659
Stim-HIV+	46.6%	1.683 (0.421, 6.725)	0.605
Stim-HIV- (ref)	33.3%	–	–
Age		1.043 (0.995, 1.093)	0.083
Tobacco use (past 3-months)			
Yes	39.3%	0.372 (0.104, 1.325)	0.127
No	47.7%	–	
hs-cTnT Concentration (Log-Normal Portion of Model)^b^			
	Estimated Mean (SD) hs-cTnT (ng/L)	Model Effects (SE)	p
Stimulant use & HIV status			
Stim+HIV+	12 (3)	0.513 (0.224)	0.031*
Stim+HIV-	3 (2)	0.130 (0.189)	0.499
Stim-HIV+	5 (3)	0.202 (0.184)	0.284
Stim-HIV- (ref)	3 (2)	–	–
Age	5 (4)	0.001 (0.007)	0.444
Tobacco use (past 3-months)			
Yes	5 (0)	−0.108 (0.171)	0.534
No	6 (4)	–	–

^a.^Probability of detectable hs-cTnT; ^b.^ Detectable values only

## Discussion

This cross-sectional study found that participant with co-occurring HIV and stimulant use had higher hs-cTnT detectability and higher estimated mean hs-cTnT than the HIV-Stim- group. These findings extend prior work linking stimulant exposure to troponin elevations and suggest that co-occurring HIV and stimulant use may identify and subgroup with greater cardiovascular vulnerability. The positive correlation between amphetamine metabolites and hs-cTnT is also consistent with this possibility, although that results was based on a small analytic subset.

High-sensitivity cardiac troponin T is a marker of myocardial injury, but detectable values are not specific to stimulant-related injury and may also be observed in people without known coronary artery disease. Depending on the clinical contact, detectable hs-cTnT may reflect chronic myocardial stress or other acute or subacute processes, including: intercurrent illness, inflammation, myocarditis, demand ischemia, renal dysfunction, acute hemodynamic stress, or structural heart disease. Interpretation is typically anchored to assay-specific reference limits (e.g., the 99^th^ percentile) [60]. In this study, most detectable values were below diagnostic thresholds, so we interpret these findings as risk-signaling rather than an intervention trigger. Because this secondary analysis did not include detailed adjudication at the time of blood draw we cannot determine the extent to which detectable hs-cTnT reflected these alternative explanations. Prior work summarizes the clinical importance of hs-cTnT in patients without coronary artery disease [61].

Results are consistent with prior research showing a dose-response between the concentration of cocaine metabolites and the concentration of cTnI in men and women [49], as well as longitudinal associations between cocaine/alcohol co-use and cTnI in women with and without HIV [50]. Taken together, findings may be consistent with higher cardiovascular risk among people who use stimulants. Whether risk counseling on these specific substances and/or including their use in CVD risk stratification would improve CVD outcomes in populations where substance use is high merits further investigation.

Further research is needed to elucidate the inflammatory and other biobehavioral mechanisms whereby co-occurring HIV and stimulant use is associated with amplified subclinical cardiac damage in men with HIV. Although cardiac troponin has been shown to be elevated during acute HIV infection, it appears to normalize in individuals receiving effective ART [62]. We have previously documented stimulant-associated elevations in soluble markers of immune activation and inflammation in sexual minority men with treated HIV who use methamphetamine.[32,63–65] In the present study, we observed significant, positive associations of TNFRI and TNFRII with higher hs-cTnT concentrations. Because multiple exploratory correlations were tested without multiplicity correction, these findings should be considered preliminary and require confirmation in larger samples. Elucidating the mechanisms whereby co-occurring HIV and stimulant use increase risk for subclinical cardiac damage is essential to guide the development of novel biobehavioral approaches to reduce CVD comorbidities. In particular, expanded efforts are needed to mitigate cardiovascular risk in those who are not ready, willing, or able to pursue stimulant abstinence.

PWH who use stimulants were also considerably more likely to have co-elevated co-occurring hs-cTnT and hs-CRP compared to the other three HIV by stimulant use groups. Although we previously demonstrated that stimulant use and HIV were independently associated with elevated hs-CRP [51], there is additional information to be gained by examining co-occurring biomarkers relevant to CVD. Previous research among people without HIV with acute coronary syndrome has shown that risk for myocardial infarction, congestive heart failure, and mortality nearly doubles with the addition of each additional biomarker (e.g., CRP, cTn, B-type natriuretic peptide) that is elevated [66,67]. Furthermore, stimulant use, primarily cocaine, has been shown to independently increase both CRP and cTn [49,50,68–71]. Taken together, elevation of hs-cTnT and hs-CRP may indicate additive risk for short- and long-term cardiac-related morbidity and mortality among men with HIV that use stimulants. Further research is needed to confirm these associations and determine whether additive biomarkers are predictive of future CVD risk in PWH.

Detectable hs-cTnT may also reflect renal dysfunction, hypertension/structural heart disease, ART-related effects, or other cardiometabolic factors [60]. Social and structural determinants that correlate with stimulant use may also contribute to cardiovascular risk. Although sensitivity models including renal function and hypertension did not materially change results, residual confounding is still possible given the cross-sectional design and sample size.

Findings from this study should be interpreted in the context of several limitations. First, the cross-sectional design precludes causal inference. Second, this secondary analysis had a fixed N and zero-inflated outcome, making the study most sensitive to detecting large effects. In particular, because most hs-cTnT values were undetectable, the detectability component of the two-part model may be vulnerable to sparse-data effects. Third, we lacked detailed clinical contxt at the time of blood draw, including information that could help distinguish chronic low-level myocardial stress from intercurrent illness or other (sub)acute causes of hs-cTnT detectability. Fourth, small group sizes limited covariate adjustment, and residual/unmeasured confounding is possible (e.g., comorbid diagnoses, other cardiovascular risk factors, hypertension and statin use, and cardiovascular disease diagnoses). Fifth, although we observed a positive association between amphetamine metabolites and hs-cTnT, this analysis was based in a small subset. Thus, it should not be over-interpreted as definitive evidence of a dose-response relationship. It also does not rule out the possibility of polysubstance use including fentanyl and the co-use of cocaine and alcohol [50]. Cocaine toxicology was unavailable, therefore, the possibility that amphetamines were reflecting correlated stimulants cannot be ruled out. Finally, the parent study enrolled cisgender men recruited primarily via advertisements on geosocial networking applications which may limit generalizability beyond sexually active men who have sex with other men in South Florida and may introduce selection bias. Additional research with large cohort studies could help elucidate existing evidence with more detail and clarity (e.g., whether specific patterns of stimulant use increase troponin more than others).

### Conclusion

In this exploratory cross-sectional sample, co-occurring HIV and stimulant use were associated with higher hs-cTnT. Additionally, amphetamine metabolite concentration was positively correlated with hs-cTnT in a small subset of participants. These findings contribute to a growing body of literature showing that stimulant use is associated with elevated hs-cTnT. The existing evidence suggests that it may be useful to assess substance use beyond alcohol and tobacco as part of cardiovascular risk assessment. Larger prospective studies with detailed clinical adjudication and more comprehensive toxicology are needed to determine whether stimulant exposure contributes to myocardial stress or injury among people with HIV.

## Supporting information

S1 Data Minimal data set. Minimal data and metadata for replication of results.
(ZIP)

## References

[1] Jespersen NA, Axelsen F, Dollerup J, Nørgaard M, Larsen CS. The burden of non-communicable diseases and mortality in people living with HIV (PLHIV) in the pre-, early- and late-HAART era. HIV Med. 2021;22(6):478–90. doi: 10.1111/hiv.13077 33645000 PMC8247855

[2] Guaraldi G, Palella FJJ. Clinical implications of aging with HIV infection: perspectives and the future medical care agenda. AIDS. 2017;31(S129). doi: 10.1097/QAD.0000000000001478 28471943

[3] Harris TG, Rabkin M, El-Sadr WM. Achieving the fourth 90: healthy aging for people living with HIV. AIDS. 2018;32(12):1563–9. doi: 10.1097/QAD.0000000000001870 29762172 PMC6082594

[4] Patel P, Rose CE, Collins PY, Nuche-Berenguer B, Sahasrabuddhe VV, Peprah E, et al. Noncommunicable diseases among HIV-infected persons in low-income and middle-income countries: a systematic review and meta-analysis. AIDS. 2018;32 Suppl 1(Suppl 1):S5–20. doi: 10.1097/QAD.0000000000001888 29952786 PMC6380891

[5] Paisible A-L, Chang C-CH, So-Armah KA, Butt AA, Leaf DA, Budoff M, et al. HIV infection, cardiovascular disease risk factor profile, and risk for acute myocardial infarction. J Acquir Immune Defic Syndr. 2015;68(2):209–16. doi: 10.1097/QAI.0000000000000419 25588033 PMC4441201

[6] Freiberg MS, Chang C-CH, Kuller LH, Skanderson M, Lowy E, Kraemer KL, et al. HIV infection and the risk of acute myocardial infarction. JAMA Intern Med. 2013;173(8):614–22. doi: 10.1001/jamainternmed.2013.3728 23459863 PMC4766798

[7] Feinstein MJ, Steverson AB, Ning H, Pawlowski AE, Schneider D, Ahmad FS, et al. Adjudicated heart failure in HIV-infected and uninfected men and women. J Am Heart Assoc. 2018;7(21):e009985. doi: 10.1161/JAHA.118.009985 30571387 PMC6404176

[8] Feinstein MJ, Hsue PY, Benjamin LA, Bloomfield GS, Currier JS, Freiberg MS, et al. Characteristics, prevention, and management of cardiovascular disease in people living with HIV: a scientific statement from the American heart association. Circulation. 2019;140(2):e98–124. doi: 10.1161/CIR.0000000000000695 31154814 PMC7993364

[9] Shah ASV, Stelzle D, Lee KK, Beck EJ, Alam S, Clifford S, et al. Global burden of atherosclerotic cardiovascular disease in people living with HIV: systematic review and meta-analysis. Circulation. 2018;138(11):1100–12. doi: 10.1161/CIRCULATIONAHA.117.033369 29967196 PMC6221183

[10] Mensah GA, Sampson UK, Roth GA, Forouzanfar MH, Naghavi M, Murray CJ. Mortality from cardiovascular diseases in sub-Saharan Africa, 1990–2013: a systematic analysis of data from the Global Burden of Disease Study 2013. Cardiovasc J Afr. 2015;26:S6-10. doi: 10.5830/CVJA-2015-036 PMC4557490 25962950

[11] Palella FJJ, Delaney KM, Moorman AC, Loveless MO, Fuhrer J, Satten GA. Declining morbidity and mortality among patients with advanced human immunodeficiency virus infection. N Engl J Med. 2009. doi: 10.1056/NEJM199803263381301 9516219

[12] Hsue PY, Waters DD. Time to recognize HIV infection as a major cardiovascular risk factor. Circulation. 2018;138(11):1113–5. doi: 10.1161/CIRCULATIONAHA.118.036211 30354392 PMC8063774

[13] Kearns A, Gordon J, Burdo TH, Qin X. HIV-1-associated atherosclerosis: unraveling the missing link. J Am Coll Cardiol. 2017;69(25):3084–98. doi: 10.1016/j.jacc.2017.05.012 28641798 PMC5512584

[14] Nou E, Lo J, Hadigan C, Grinspoon SK. Pathophysiology and management of cardiovascular disease in patients with HIV. Lancet Diabetes Endocrinol. 2016;4(7):598–610. doi: 10.1016/S2213-8587(15)00388-5 26873066 PMC4921313

[15] Hsue PY, Waters DD. HIV infection and coronary heart disease: mechanisms and management. Nat Rev Cardiol. 2019;16(12):745–59. doi: 10.1038/s41569-019-0219-9 31182833 PMC8015945

[16] Ballocca F, D’Ascenzo F, Gili S, Grosso Marra W, Gaita F. Cardiovascular disease in patients with HIV. Trends Cardiovasc Med. 2017;27(8):558–63. doi: 10.1016/j.tcm.2017.06.005 28779949

[17] Vachiat A, McCutcheon K, Tsabedze N, Zachariah D, Manga P. HIV and ischemic heart disease. J Am Coll Cardiol. 2017;69(1):73–82. doi: 10.1016/j.jacc.2016.09.979 28057253

[18] Tadrous M, Shakeri A, Chu C, Watt J, Mamdani MM, Juurlink DN, et al. Assessment of stimulant use and cardiovascular event risks among older adults. JAMA Netw Open. 2021;4(10):e2130795. doi: 10.1001/jamanetworkopen.2021.30795 34694389 PMC8546494

[19] Havakuk O, Rezkalla SH, Kloner RA. The cardiovascular effects of cocaine. J Am Coll Cardiol. 2017;70(1):101–13. doi: 10.1016/j.jacc.2017.05.014 28662796

[20] Paratz ED, Cunningham NJ, MacIsaac AI. The Cardiac Complications of Methamphetamines. Heart Lung Circ. 2016;25(4):325–32. doi: 10.1016/j.hlc.2015.10.019 26706652

[21] Schürer S, Klingel K, Sandri M, Majunke N, Besler C, Kandolf R, et al. Clinical characteristics, histopathological features, and clinical outcome of methamphetamine-associated cardiomyopathy. JACC Heart Fail. 2017;5(6):435–45. doi: 10.1016/j.jchf.2017.02.017 28571597

[22] Gan WQ, Buxton JA, Scheuermeyer FX, Palis H, Zhao B, Desai R, et al. Risk of cardiovascular diseases in relation to substance use disorders. Drug Alcohol Depend. 2021;229(Pt A):109132. doi: 10.1016/j.drugalcdep.2021.109132 34768052

[23] Mladěnka P, Applová L, Patočka J, Costa VM, Remiao F, Pourová J, et al. Comprehensive review of cardiovascular toxicity of drugs and related agents. Med Res Rev. 2018;38(4):1332–403. doi: 10.1002/med.21476 29315692 PMC6033155

[24] Chelikam N, Vyas V, Dondapati L, Iskander B, Patel G, Jain S, et al. Epidemiology, burden, and association of substance abuse amongst patients with cardiovascular disorders: national cross-sectional survey study. Cureus. 2022;14(7):e27016. doi: 10.7759/cureus.27016 35989848 PMC9386401

[25] Brgdar A, Gharbin J, Elawad A, Yi J, Sanchez J, Bishaw A, et al. Effects of substance use disorder on in-hospital outcomes of young patients presenting with a cardiovascular event: a nationwide analysis. Cureus. 2022;14(3):e22737. doi: 10.7759/cureus.22737 35386479 PMC8969757

[26] Mimiaga MJ, Reisner SL, Grasso C, Crane HM, Safren SA, Kitahata MM, et al. Substance use among HIV-infected patients engaged in primary care in the United States: findings from the Centers for AIDS Research Network of Integrated Clinical Systems cohort. Am J Public Health. 2013;103(8):1457–67. doi: 10.2105/AJPH.2012.301162 23763417 PMC3752382

[27] Rosen MI, Black AC, Arnsten JH, Goggin K, Remien RH, Simoni JM, et al. Association between use of specific drugs and antiretroviral adherence: findings from MACH 14. AIDS Behav. 2013;17(1):142–7. doi: 10.1007/s10461-011-0124-7 22246513 PMC3549004

[28] NIH Office of AIDS Research. Substance use disorders and HIV. Considerations for antiretroviral use in special patient populations. 2021. https://clinicalinfo.hiv.gov/en/guidelines/hiv-clinical-guidelines-adult-and-adolescent-arv/substance-use-disorders-and-hiv

[29] Centers for Disease Control and Prevention. Behavioral and clinical characteristics of persons with diagnosed HIV infection—Medical monitoring project, United States 2021 cycle (June 2021–May 2022). Centers for Disease Control and Prevention; 2019.

[30] Philbin MM, Greene ER, Martins SS, LaBossier NJ, Mauro PM. Medical, nonmedical, and illegal stimulant use by sexual identity and gender. Am J Prev Med. 2020;59(5):686–96. doi: 10.1016/j.amepre.2020.05.025 32981768 PMC7577928

[31] Ellis RJ, Childers ME, Cherner M, Lazzaretto D, Letendre S, Grant I, et al. Increased human immunodeficiency virus loads in active methamphetamine users are explained by reduced effectiveness of antiretroviral therapy. J Infect Dis. 2003;188(12):1820–6. doi: 10.1086/379894 14673760

[32] Carrico AW, Johnson MO, Morin SF, Remien RH, Riley ED, Hecht FM, et al. Stimulant use is associated with immune activation and depleted tryptophan among HIV-positive persons on anti-retroviral therapy. Brain Behav Immun. 2008;22(8):1257–62. doi: 10.1016/j.bbi.2008.07.010 18703133 PMC2783360

[33] Massanella M, Gianella S, Schrier R, Dan JM, Pérez-Santiago J, Oliveira MF, et al. Methamphetamine use in HIV-infected individuals affects T-cell function and viral outcome during suppressive antiretroviral therapy. Sci Rep. 2015;5:13179. doi: 10.1038/srep13179 26299251 PMC4547398

[34] Baum MK, Rafie C, Lai S, Sales S, Page B, Campa A. Crack-cocaine use accelerates HIV disease progression in a cohort of HIV-positive drug users. J Acquir Immune Defic Syndr. 2009;50(1):93–9. doi: 10.1097/QAI.0b013e3181900129 19295339

[35] Cook JA, Burke-Miller JK, Cohen MH, Cook RL, Vlahov D, Wilson TE, et al. Crack cocaine, disease progression, and mortality in a multicenter cohort of HIV-1 positive women. AIDS. 2008;22(11):1355–63. doi: 10.1097/QAD.0b013e32830507f2 18580615 PMC2645902

[36] Marston S, Zamora JE. Troponin structure and function: a view of recent progress. J Muscle Res Cell Motil. 2020;41(1):71–89. doi: 10.1007/s10974-019-09513-1 31030382 PMC7109197

[37] Anderson PA, Greig A, Mark TM, Malouf NN, Oakeley AE, Ungerleider RM, et al. Molecular basis of human cardiac troponin T isoforms expressed in the developing, adult, and failing heart. Circ Res. 1995;76(4):681–6. doi: 10.1161/01.res.76.4.681 7534662

[38] Kelley WE, Januzzi JL, Christenson RH. Increases of cardiac troponin in conditions other than acute coronary syndrome and heart failure. Clin Chem. 2009;55(12):2098–112. doi: 10.1373/clinchem.2009.130799 19815610

[39] Potter JM, Hickman PE, Cullen L. Troponins in myocardial infarction and injury. Aust Prescr. 2022;45(2):53–7. doi: 10.18773/austprescr.2022.006 35592367 PMC9081942

[40] Neumann JT, Havulinna AS, Zeller T, Appelbaum S, Kunnas T, Nikkari S, et al. Comparison of three troponins as predictors of future cardiovascular events--prospective results from the FINRISK and BiomaCaRE studies. PLoS One. 2014;9(3):e90063. doi: 10.1371/journal.pone.0090063 24594734 PMC3942371

[41] Blankenberg S, Salomaa V, Makarova N, Ojeda F, Wild P, Lackner KJ, et al. Troponin I and cardiovascular risk prediction in the general population: the BiomarCaRE consortium. Eur Heart J. 2016;37(30):2428–37. doi: 10.1093/eurheartj/ehw172 27174290 PMC4982535

[42] McCarthy C, Li S, Wang TY, Raber I, Sandoval Y, Smilowitz NR, et al. Implementation of high-sensitivity cardiac troponin assays in the United States. J Am College Cardiol 2023;81:207–19. doi: 10.1016/j.jacc.2022.10.017 PMC10037558 36328155

[43] Raber I, McCarthy CP, Januzzi JL Jr. A test in context: interpretation of high-sensitivity cardiac troponin assays in different clinical settings. J Am Coll Cardiol. 2021;77(10):1357–67. doi: 10.1016/j.jacc.2021.01.011 33706879

[44] Rahman F, Zhang Z, Zhao D, Budoff MJ, Palella FJ, Witt MD, et al. Association of high-sensitivity troponin with cardiac CT angiography evidence of myocardial and coronary disease in a primary prevention cohort of men: results from MACS. J Appl Lab Med. 2019;4(3):355–69. doi: 10.1373/jalm.2018.028860 31659073 PMC7085121

[45] Chattranukulchai P, Vassara M, Siwamogsatham S, Buddhari W, Tumkosit M, Ketloy C, et al. High-sensitivity troponins and subclinical coronary atherosclerosis evaluated by coronary calcium score among older asians living with well-controlled human immunodeficiency virus. Open Forum Infect Dis. 2023;10(7):ofad234. doi: 10.1093/ofid/ofad234 37404953 PMC10317471

[46] Jefferson A, Haberlen S, Plankey M, Piggott D, Brown T, Palella FJ JR, et al. Associations between high sensitivity troponin levels, HIV serostatus and cardiac MRI measures. J Am College Cardiol. 2022;79(9):1630. doi: 10.1016/s0735-1097(22)02621-3

[47] Ahmed HA, Mohamed J, Akuku IG, Lee KK, Alam SR, Perel P, et al. Cardiovascular risk factors and markers of myocardial injury and inflammation in people living with HIV in Nairobi, Kenya: a pilot cross-sectional study. BMJ Open. 2022;12(6):e062352. doi: 10.1136/bmjopen-2022-062352 35667720 PMC9171254

[48] Foster P, Sokoll L, Li J, Gerstenblith G, Fishman EK, Kickler T, et al. Circulating levels of cardiac troponin T are associated with coronary noncalcified plaque burden in HIV-infected adults: a pilot study. Int J STD AIDS. 2019;30(3):223–30. doi: 10.1177/0956462418800873 30381028 PMC6921494

[49] Riley ED, Hsue PY, Vittinghoff E, Wu AHB, Coffin PO, Moore PK, et al. Higher prevalence of detectable troponin I among cocaine-users without known cardiovascular disease. Drug Alcohol Depend. 2017;172:88–93. doi: 10.1016/j.drugalcdep.2016.11.039 28157591 PMC5464776

[50] Riley ED, Vittinghoff E, Wu AHB, Coffin PO, Hsue PY, Kazi DS, et al. Impact of polysubstance use on high-sensitivity cardiac troponin I over time in homeless and unstably housed women. Drug Alcohol Depend. 2020;217:108252. doi: 10.1016/j.drugalcdep.2020.108252 32919207 PMC7873814

[51] Cherenack EM, Chavez JV, Martinez C, Hirshfield S, Balise R, Horvath KJ, et al. Stimulant use, HIV, and immune dysregulation among sexual minority men. Drug Alcohol Depend. 2023;251:110942. doi: 10.1016/j.drugalcdep.2023.110942 37651812 PMC10544798

[52] Redwood Toxicology Laboratory. ICUP® drug screen. 2023. https://www.redwoodtoxicology.com/devices/doa_icup

[53] WHO ASSIST Working Group. The alcohol, smoking and substance involvement screening test (ASSIST): development, reliability and feasibility. Addiction. 2002;97(9):1183–94. doi: 10.1046/j.1360-0443.2002.00185.x 12199834

[54] Orasure Technologies, Inc. OraQuick advanced HIV 1/2 antibody test. Bethlehem, Pennsylvania. 2006.

[55] Verhofstede C, Van Wanzeele F, Reynaerts J, Mangelschots M, Plum J, Fransen K. Viral load assay sensitivity and low level viremia in HAART treated HIV patients. J Clin Virol. 2010;47(4):335–9. doi: 10.1016/j.jcv.2010.01.008 20138803

[56] Li Y, Zhong X, Cheng G, Zhao C, Zhang L, Hong Y, et al. Hs-CRP and all-cause, cardiovascular, and cancer mortality risk: a meta-analysis. Atherosclerosis. 2017;259:75–82. doi: 10.1016/j.atherosclerosis.2017.02.003 28327451

[57] Bassuk SS, Rifai N, Ridker PM. High-sensitivity C-reactive protein: clinical importance. Curr Probl Cardiol. 2004;29(8):439–93. doi: 10.1016/s0146-2806(04)00074-x 15258556

[58] Rifai N, Ridker PM. High-sensitivity C-reactive protein: a novel and promising marker of coronary heart disease. Clin Chem. 2001;47(3):403–11. doi: 10.1093/clinchem/47.3.403 11238289

[59] Sabatine MS, Morrow DA, de Lemos JA, Gibson CM, Murphy SA, Rifai N, et al. Multimarker approach to risk stratification in non-ST elevation acute coronary syndromes. Circulation. 2002;105(15):1760–3. doi: 10.1161/01.cir.0000015464.18023.0a 11956114

[60] Thygesen K, Alpert JS, Jaffe AS, Chaitman BR, Bax JJ, Morrow DA, et al. Fourth universal definition of myocardial infarction (2018). Circulation. 2018;138:e618-51. doi: 10.1161/CIR.0000000000000617 30571511

[61] Askin L, Tanriverdi O, Turkmen S. Clinical importance of high- sensitivity troponin T in patients without coronary artery disease. North Clin Istanb. 2020;7(3):305–10. doi: 10.14744/nci.2019.71135 32478307 PMC7251271

[62] Schuster C, Mayer FJ, Wohlfahrt C, Marculescu R, Skoll M, Strassl R, et al. Acute HIV infection results in subclinical inflammatory cardiomyopathy. J Infect Dis. 2018;218(3):466–70. doi: 10.1093/infdis/jiy183 29608697

[63] Carrico AW, Cherenack EM, Roach ME, Riley ED, Oni O, Dilworth SE, et al. Substance-associated elevations in monocyte activation among methamphetamine users with treated HIV infection. AIDS. 2018;32(6):767–71. doi: 10.1097/QAD.0000000000001751 29369159 PMC5912167

[64] Carrico AW, Flentje A, Kober K, Lee S, Hunt P, Riley ED, et al. Recent stimulant use and leukocyte gene expression in methamphetamine users with treated HIV infection. Brain Behav Immun. 2018;71:108–15. doi: 10.1016/j.bbi.2018.04.004 29679637 PMC6003871

[65] Grosgebauer K, Salinas J, Sharkey M, Roach M, Pallikkuth S, Dilworth SE, et al. Psychosocial correlates of monocyte activation and HIV persistence in methamphetamine users. J Neuroimmune Pharmacol. 2019;14(1):16–22. doi: 10.1007/s11481-018-9797-2 30046962 PMC6347547

[66] Sabatine MS, Morrow DA, de Lemos JA, Gibson CM, Murphy SA, Rifai N, et al. Multimarker approach to risk stratification in non-ST elevation acute coronary syndromes: simultaneous assessment of troponin I, C-reactive protein, and B-type natriuretic peptide. Circulation. 2002;105(15):1760–3. doi: 10.1161/01.cir.0000015464.18023.0a 11956114

[67] Morrow DA, Rifai N, Antman EM, Weiner DL, McCabe CH, Cannon CP, et al. C-reactive protein is a potent predictor of mortality independently of and in combination with troponin T in acute coronary syndromes: a TIMI 11A substudy. Thrombolysis in Myocardial Infarction. J Am Coll Cardiol. 1998;31(7):1460–5. doi: 10.1016/s0735-1097(98)00136-3 9626820

[68] McCord J, Jneid H, Hollander JE, de Lemos JA, Cercek B, Hsue P, et al. Management of cocaine-associated chest pain and myocardial infarction. Circulation. 2008;117(14):1897–907. doi: 10.1161/circulationaha.107.188950 18347214

[69] Moore P, Lynch KL, Wu AHB, Ma Y, Scherzer R, Li D, et al. Association of cocaine and amphetamine use with troponin I concentrations. J Am College Cardiol. 2017;69(11):281. doi: 10.1016/s0735-1097(17)33670-7

[70] Wang T-Y, Lu R-B, Lee S-Y, Chang Y-H, Chen S-L, Tsai T-Y, et al. Association between inflammatory cytokines, executive function, and substance use in patients with opioid use disorder and amphetamine-type stimulants use disorder. Int J Neuropsychopharmacol. 2023;26(1):42–51. doi: 10.1093/ijnp/pyac069 36181736 PMC9850661

[71] Nazari A, Zahmatkesh M, Mortaz E, Hosseinzadeh S. Effect of methamphetamine exposure on the plasma levels of endothelial-derived microparticles. Drug Alcohol Depend. 2018;186:219–25. doi: 10.1016/j.drugalcdep.2018.02.015 29609134

